# Evaluation of Computational Methodologies for Accurate Prediction of Wall Shear Stress and Turbulence Parameters in a Patient-Specific Aorta

**DOI:** 10.3389/fbioe.2022.836611

**Published:** 2022-03-24

**Authors:** Emily Louise Manchester, Selene Pirola, Mohammad Yousuf Salmasi, Declan P. O’Regan, Thanos Athanasiou, Xiao Yun Xu

**Affiliations:** ^1^ Department of Chemical Engineering, Imperial College London, London, United Kingdom; ^2^ Department of Surgery and Cancer, Imperial College London, St Mary’s Hospital, London, United Kingdom; ^3^ MRC London Institute of Medical Sciences, Imperial College London, Hammersmith Hospital, London, United Kingdom

**Keywords:** aorta, computational fluid dynamics, magnetic resonance imaging, laminar, turbulence, large-eddy simulation, wall shear stress, viscous energy loss

## Abstract

**Background:** Recent studies suggest that blood flow in main arteries is intrinsically disturbed, even under healthy conditions. Despite this, many computational fluid dynamics (CFD) analyses of aortic haemodynamics make the assumption of laminar flow, and best practices surrounding appropriate modelling choices are lacking. This study aims to address this gap by evaluating different modelling and post-processing approaches in simulations of a patient-specific aorta.

**Methods:** Magnetic resonance imaging (MRI) and 4D flow MRI from a patient with aortic valve stenosis were used to reconstruct the aortic geometry and derive patient-specific inlet and outlet boundary conditions. Three different computational approaches were considered based on assumed laminar or assumed disturbed flow states including low-resolution laminar (LR-Laminar), high-resolution laminar (HR-Laminar) and large-eddy simulation (LES). Each simulation was ran for 30 cardiac cycles and post-processing was conducted on either the final cardiac cycle, or using a phase-averaged approach which utilised all 30 simulated cycles. Model capabilities were evaluated in terms of mean and turbulence-based parameters.

**Results:** All simulation types, regardless of post-processing approach could correctly predict velocity values and flow patterns throughout the aorta. Lower resolution simulations could not accurately predict gradient-derived parameters including wall shear stress and viscous energy loss (largest differences up to 44.6% and 130.3%, respectively), although phase-averaging these parameters improved predictions. The HR-Laminar simulation produced more comparable results to LES with largest differences in wall shear stress and viscous energy loss parameters up to 5.1% and 11.6%, respectively. Laminar-based parameters were better estimated than turbulence-based parameters.

**Conclusion:** Our findings suggest that well-resolved laminar simulations can accurately predict many laminar-based parameters in disturbed flows, but there is no clear benefit to running a HR-Laminar simulation over an LES simulation based on their comparable computational cost. Additionally, post-processing “typical” laminar simulation results with a phase-averaged approach is a simple and cost-effective way to improve accuracy of lower-resolution simulation results.

## 1 Introduction

Patient-specific computational fluid dynamic (CFD) analysis has been widely adopted in the biomedical community. Simulation outputs can be used in several ways including evaluation and design of medical devices; informing clinical decisions; and understanding disease progressions to name only a few (Saloner et al., 2006; Ge et al., 2008; Borazjani et al., 2010). Haemodynamic parameters, such as wall shear stress (WSS), are used to investigate the mechanical shearing force exerted by blood flow on the inner arterial wall and thereby endothelial cells which are in direct contact with blood. WSS is one of the factors which determine endothelium homeostasis and WSS extremes affect endothelial cell response, promoting vascular remodelling and pathologies (Cunningham and Gotlieb, 2005; Dolan et al., 2013). More specifically, prolonged exposure to high WSS is associated with aortic growth, extracellular matrix dysregulation and elastic fibre degeneration (Guzzardi et al., 2015; Bollache et al., 2018), and a recent study into ascending aortic aneurysms concluded that high WSS is associated with wall degradation in the ascending aorta (Salmasi et al., 2021). Fluctuations in WSS which occur in disturbed flows induce endothelial dysfunction (Davies et al., 1986; Chiu and Chien, 2011). Similarly, laminar and fluctuating viscous shear stresses within a fluid can be used to evaluate energy losses and haemolysis (Yen et al., 2014). Wall and viscous shear stresses are important biomarkers and it is therefore crucial that CFD models are accurate and simulation outputs are correctly processed.

There are numerous CFD studies into aortic haemodynamics, however the majority of these studies made the assumption of laminar flow. In recent years, there have been studies of aortic flows which do not assume laminarity and have shown disturbances to be present in aortas with and without pathologic conditions (Lantz et al., 2012; Lantz et al., 2013; Miyazaki et al., 2017; Xu et al., 2018; Xu et al., 2020; Manchester et al., 2021). A recent study considered flow in the healthy aorta, deducing that physiological blood flow is non-laminar and displays blood flow disturbances (Saqr et al., 2020). Recent literature suggests that the widely accepted theory of laminar flow in large arteries by the scientific community may need to be revisited. A computational study into aorta flows compared modelling capabilities of different simulation types including laminar, large-eddy simulation (LES) and the renormalisation group (RNG) *k* − *ϵ* model, although it was uncertain which modelling approach performed the best (Miyazaki et al., 2017). Lancellotti et al. (2017) considered a patient-specific stenotic carotid artery which produced a high shear and transitional flow. LES simulations with various Sigma and Smagorinsky-type subgrid-scale (SGS) models were compared to a higher-resolution simulation without SGS modelling, akin to direct numerical simulation (DNS). The static Sigma model performed best and was more robust than dynamic and mixed Sigma models. All Smagorinsky-type models were unstable and caused simulation blow-up. Similar to Lancellotti et al. (2017), Mancini et al. (2019) compared LES simulations using static Smagorinsky, dynamic Smagorinsky and static Sigma SGS models against an under-resolved DNS simulation in a stenotic carotid artery. They found that both static Sigma and dynamic Smagorinsky models could produce reliable results under pulsatile conditions, and the static Sigma model had lower computational costs than dynamic Smagorinsky. Andersson and Karlsson (2021) evaluated model-related errors in LES simulations of an aortic coarctation model. Turbulence-related tensor characteristic sensitivities to spatiotemporal resolution and phase-averaging sample size were assessed. It was found that phase-averaging errors associated with too few cardiac cycles could outweigh spatiotemporal resolution errors. Xu et al. (2020) compared laminar and LES simulations of patient-specific aortas with dilation and different aortic valve morphologies. Large-scale flow parameters were in good agreement although larger differences occurred in disturbed flow regions. Despite progression in our understanding of blood flow states, as well as efforts towards modelling guidelines in disturbed cardiovascular flows, it is still unclear which modelling approach should be selected in aortic computational simulations. This is especially true considering the high computational costs associated with LES; which are not always practicable. Understanding the capabilities of other simulation approaches without turbulence models (e.g., laminar simulation-types) in predicting both laminar and turbulence-based parameters of interest will help inform appropriate model selection in future studies.

The objective of this research is to evaluate the performance of different computational approaches used in simulations of patient-specific aortic flow. A patient with aortic valve stenosis was selected for this study as it showed blood flow disturbances (Manchester et al., 2021) and is thus expected to provide a challenging case for the various computational approaches. Three simulations are conducted including low-resolution laminar, high-resolution laminar and large-eddy simulation, and detailed comparisons are made in terms of both laminar and turbulence parameters.

## 2 Materials and Methods

### 2.1 Data Acquisition and Magnetic Resonance Image Processing

A patient-with aortic valve stenosis was recruited from St Bartholomew’s Hospital (London, United Kingdom) and underwent cardiac magnetic resonance imaging (MRI) and 4D flow MRI at Hammersmith Hospital (London, United Kingdom). The study received ethical approval from the Health Research Authority and Regional Ethics Committee (17/NI/0160) and was sponsored by the Imperial College London Joint Research and Compliance Office, as defined under the sponsorship requirements of the Research Governance Framework (2005). MRI was used to reconstruct the aorta geometry consisting of the ascending aorta down to the descending thoracic aorta and inclusive of the three supra-aortic branches. At the inlet, 4D flow MRI was used to derive a 3D velocity profile over a cardiac cycle. At the three-branch outlets the pressure-based 3-element Windkessel model is applied, with parameters determined using flow waveforms derived from 4D flow MRI and central aortic pressure measurements acquired using a brachial cuff. At the descending thoracic aorta outlet, a mass flow waveform is prescribed based on a fixed flow-split which was estimated from 4D flow MRI. The arterial wall is assumed rigid with a no-slip boundary condition. Full details on data acquisition and image processing can be found in our previous study (Manchester et al., 2021).

### 2.2 Computational Approaches

Two different computational approaches were considered including laminar and large-eddy simulation (LES). For a true laminar state of flow, competently executed laminar simulations can provide accurate results which rival measurements. Laminar simulations do not explicitly include a turbulence model meaning they are computationally less demanding compared to other simulation approaches. For disturbed flows, LES is better suited owing to its capabilities in modelling laminar, transitional and turbulence features. An implicit LES utilises the computational mesh to distinguish between different length scales of the flow whereby eddies larger than the mesh are directly resolved and eddies smaller than the mesh are accounted for using a subgrid-scale model. In this study, the wall-adapting local eddy-viscosity (WALE) subgrid-scale model is used (Nicoud and Ducros, 1999), with model coefficient *C*_*w*_ = 0.325. The LES methodology was previously validated in both idealised and patient-specific settings, and full details on the LES implementation can be found in our previous publications (Manchester and Xu, 2020; Manchester et al., 2021).

In this study, three different simulation types were considered including low-resolution laminar (LR-Laminar), high-resolution laminar (HR-Laminar) and LES. The naming conventions (LR- and HR-Laminar) refer to both spatial discretisation and temporal discretisation resolutions; where LR-Laminar indicates a lower resolution simulation with coarser mesh and larger time-step, and HR-Laminar indicates a higher resolution simulation with finer mesh and smaller time-step. In the case of laminar-type simulations of non-laminar flows, the length and time-scales of flow which are greater than the spatial and temporal discretisation of the domain are directly resolved. This means that the HR-Laminar simulation is effectively an implicit LES simulation without a subgrid-scale model, meaning the large-scale turbulence features are directly resolved and the influence of the small-scales are not included. The HR-Laminar simulation can essentially be viewed as an unresolved or quasi-direct numerical simulation. The LR-Laminar simulation is designed to be representative of “typical” laminar aortic simulations reported in previous studies (Cheng et al., 2016; Pirola et al., 2017) and both mesh size and time-step are selected accordingly. Similarly to the HR-Laminar simulation, large-scale turbulence features are resolved and the influence of the small-scales are not included. Therefore, in a coarser mesh (LR-Laminar), fewer turbulence scales are resolved.

#### 2.2.1 Computational Mesh and Time-Step

A structured meshing approach was used with meshes generated in ANSYS ICEM (v17.0, ANSYS Inc., Canonsburg, PA). Octagonal multi-block structures were used for greater user control and to allow proper near wall treatment, ensuring *y* ^+^ < 1. The LES and HR-Laminar simulations used the same mesh and time-step, and LR-Laminar used a coarser mesh and larger time-step. For all meshes, the default ICEM quality metric was above 0.35. The LES and HR-Laminar mesh has a quality metric greater than 0.7 for 97% of the fluid domain, and the LR-Laminar mesh quality metric is greater than 0.7 for 96% of the fluid domain. Full mesh and time-step details used in the different simulations included in this study are provided in Table 1. Mesh and time-step sensitivity tests were conducted at peak systole. The LR-Laminar mesh used in this study consists of 1.8 million cells and a mesh sensitivity test was performed by refining the mesh by a factor of 1.3 in all directions, resulting in a finer mesh of 3.9 million cells. In typical laminar simulations of aorta flows, turbulence-based parameters are not included in sensitivity tests, therefore only mean kinetic energy and mean wall shear stress were considered in the mesh sensitivity analysis. Compared to the 3.9 million cell mesh, differences between 1.2% and 3.7% were observed. For the LES and HR-Laminar mesh, mean and turbulence-based parameters were converged including mean kinetic energy, turbulence kinetic energy, mean wall shear stress and turbulent wall shear stress. Full details on the mesh sensitivity can be found in Manchester et al. (2021). Two-point correlations were also used to evaluate streamwise and radial spatial resolutions in regions of elevated turbulence. The two-point correlation estimates the number of cells which resolve the largest turbulence scales and it is recommended that 8 cells or more are sufficient for LES simulations (Davidson, 2009). The mesh used for the LES and HR-Laminar simulation used at least 20 cells to resolve the largest scales in the streamwise directions and 8 cells in the radial directions, suggesting a well resolved mesh. The complete two-point correlation results can be found in Supplementary Material.

**TABLE 1:** Numerical, mesh and time-step details.

Simulation Type	Subgrid-scale model	Number of cells [million]	Mean cell Height [mm]	First wall- adjacent cell height [mm]	Number of cells in boundary layer	Time-step [ms]
LR-Laminar	None	1.8	1.06	0.1	10	1
HR-Laminar	None	7.4	0.53	0.01	16	0.2
LES	WALE	7.4	0.53	0.01	16	0.2

#### 2.2.2 Numerical Details

Simulations were performed in OpenFOAM and ran on Cirrus UK National Tier-2 HPC with 216 cores. The fluid was assumed incompressible and Newtonian, with fluid properties representative of blood (*ρ* = 1060 *kg*/*m* ^3^ and *μ* = 0.0035 *Pa* *s*). Temporal discretisation was achieved using a second order implicit backwards Euler scheme. For LES and HR-Laminar simulations, spatial discretisation was achieved using a second-order central differencing scheme (Gauss) and for LR-Laminar, spatial discretisation was achieved using a bounded second-order upwind scheme. Simulations were converged to a normalised residual of 1e-5 at each time-step for velocity and pressure. Pressure and velocity coupling was achieved using the PIMPLE algorithm. 30 cardiac cycles were simulated to ensure convergence of the phase-averaged parameters, as is discussed in the following section.

### 2.3 Post-Processing

Both laminar and turbulence-related parameters are presented including velocity, wall shear stress, viscous dissipation, and turbulence kinetic energy. Parameters are calculated using two different methods based on the expected flow state. The first implements an approach typically used to post-process laminar simulations of periodic arterial flows, and the second corresponds to an approach used to post-process simulation results of unstable or turbulent flows. In the first approach, it is assumed that the flow is laminar and pulsating, meaning flow reaches a periodically steady state, i.e., that cycle-to-cycle variations do not occur. Once a sufficient number of cardiac cycles have been simulated to reach a periodic solution, the simulation is stopped and post-processing is conducted on results obtained in the final cycle only, using instantaneous parameters. This method of post-processing is used for laminar-based parameters of the LR-Laminar simulation only using the final cardiac cycle. All parameters post-processed using this approach are referred to as ILR-Laminar (instantaneous LR-Laminar).

The second approach assumes that flow is unstable, and cycle-to-cycle variations may occur. In this case, an instantaneous variable can be decomposed into phase-averaged and fluctuating components:

$$\phi \left(x,t\right)=\left\langle \phi \right\rangle \left(x,t\right)+\phi^{\prime }\left(x,t\right)$$ (1)

The phase-average operator, ⟨.⟩ acts to average a given variable at a fixed point in time (e.g., peak systole) over all simulated cardiac cycles:

$$\left\langle \phi \right\rangle \left(x,t\right)=\frac{1}{N}\overset{N-1}{\underset{n=0}{\sum }}\phi \left(x,t+nT\right)$$ (2)

where *N* is the total number of cardiac cycles, *T* the period of the cardiac cycle and *t* is a specified time within a cycle. For disturbed pulsatile flows, the phase-average provides the correct mean representation of a variable at any given time in the cardiac cycle.

The phase-averaged fluctuating component is given by the root-mean-square (RMS) of the instantaneous and phase-average variables:

$$\langle \phi^{\prime }\rangle \left(x,t\right)=\sqrt{\frac{1}{N}\overset{N-1}{\underset{n=0}{\sum }}{\left(\phi \left(x,t+nT\right)-\langle \phi \rangle \left(x,t\right)\right)}^{2}}$$ (3)

This method of post-processing is applied to the LR-Laminar, HR-Laminar and LES simulations. Integrating any variable over the full cardiac cycle results in a cycle-average, referred to as the time-average:

$$\bar{\phi }\left(x,t\right)=\frac{1}{T}\int_{0}^{T}\phi \left(x,t\right)\;dt$$ (4)

Equation 4 represents the time-average of an instantaneous variable, as used in ILR-Laminar. Replacing *ϕ* with ⟨*ϕ*⟩ gives the time-average of a phase-averaged mean variable, and replacing *ϕ* with ⟨*ϕ*′⟩ gives the time-average of a phase-averaged turbulent variable. The latter substitutions are used in LR-Laminar, HR-Laminar and LES simulations. All results presented in this paper represent the phase-average, unless indicated as ILR-Laminar. Using two common methods of post-processing allows to not only assess resolution-based performance but also understand the effects of the post-processing approach.

#### 2.3.1 Haemodynamic Parameters

Wall shear stress (WSS) is the instantaneous shearing force exerted by a fluid on the inner surface of the arterial wall. WSS can be decomposed into phase-averaged WSS, ⟨*τ*_*wall*_⟩, using Eq. 2, and decomposed into turbulent-WSS, $\left\langle \tau_{wall}^{\prime }\right\rangle$ , using Eq. 3. Applying Eq. 4 to the phase-averaged WSS gives the time-averaged wall shear stress (TAWSS). Similarly, applying Eq. 4 to the turbulent-WSS gives the turbulent time-averaged WSS (turbulent-TAWSS). All the wall shear stress-related parameters used in this study are provided in Table 2. Oscillatory shear index (OSI) is given by:

$$OSI=0.5\left(1-\frac{\left|\int_{0}^{T}\langle WSS\rangle dt\right|}{\int_{0}^{T}|\langle WSS\rangle |dt}\right)$$ (5)

**TABLE 2:** Wall shear stress parameter definitions.

Parameter	Equation
WSS	$\tau_{wall}\left(x,t\right)=\mu \frac{\partial u\left(x,t\right)}{\partial n\left(x\right)}$
Phase-averaged WSS	$\left\langle \tau_{wall}\right\rangle \left(x,t\right)=\frac{1}{N}\sum_{n=0}^{N-1}\tau_{wall}\left(x,t+nT\right)$
Turbulent WSS	$\left\langle \tau_{wall}^{\prime }\right\rangle \left(x,t\right)=\sqrt{\frac{1}{N}\sum_{n=0}^{N-1}{\left(\tau_{wall}\left(x,t+nT\right)-\left\langle \tau_{wall}\right\rangle \left(x,t\right)\right)}^{2}}$
TAWSS	$\bar{\left\langle \tau_{wall}\right\rangle }\left(x,t\right)=\frac{1}{T}\int_{0}^{T}\left\langle \tau_{wall}\right\rangle \left(x,t\right)\;dt$
Turbulent-TAWSS	$\bar{\left\langle \tau_{wall}^{\prime }\right\rangle }\left(x,t\right)=\frac{1}{T}\int_{0}^{T}\left\langle \tau_{wall}^{\prime }\right\rangle \left(x,t\right)\;dt$

For ILR-Laminar, ⟨*WSS*⟩ is replaced with the instantaneous WSS.

Turbulence kinetic energy (TKE) is associated with eddies in disturbed flows and can be used to quantify the level of turbulence. TKE is calculated from fluctuating velocity components:

$$TKE=\frac{\rho }{2}\underset{i}{\sum }{u_{i}^{\prime }}^{2}\;\left[Pa\right]$$ (6)

where *ρ* is the fluid density and *i* = 1, 2, 3.

Viscous dissipation is used to quantify frictional losses, which is a measure of the work done by a fluid on its adjacent layers due to shearing forces. The rate of laminar viscous energy loss can be estimated from the velocity gradient tensor by integrating the viscous dissipation function over the aortic volume:

$$\overset{.}{EL}=\frac{\mu }{2}\int_{V}\underset{i,j}{\sum }{\left(\frac{\partial \langle u_{i}\rangle }{\partial x_{j}}+\frac{\partial \langle u_{j}\rangle }{\partial x_{i}}\right)}^{2}\;dV\;\left[W\right]$$ (7)

Similarly, the rate of turbulent viscous energy loss is calculated using the fluctuating velocity gradient tensor:

$$\overset{.}{EL^{\prime }}=\frac{\mu }{2}\int_{V}\underset{i,j}{\sum }{\left(\frac{\partial u_{i}^{\prime }}{\partial x_{j}}+\frac{\partial u_{j}^{\prime }}{\partial x_{i}}\right)}^{2}\;dV\;\left[W\right]$$ (8)

Integrating the rates of laminar and turbulent dissipation over a cardiac cycle gives the net laminar viscous energy loss and net turbulent viscous energy loss per cardiac cycle, respectively.

#### 2.3.2 Analysis and Comparison of Results

**FIGURE 1:**
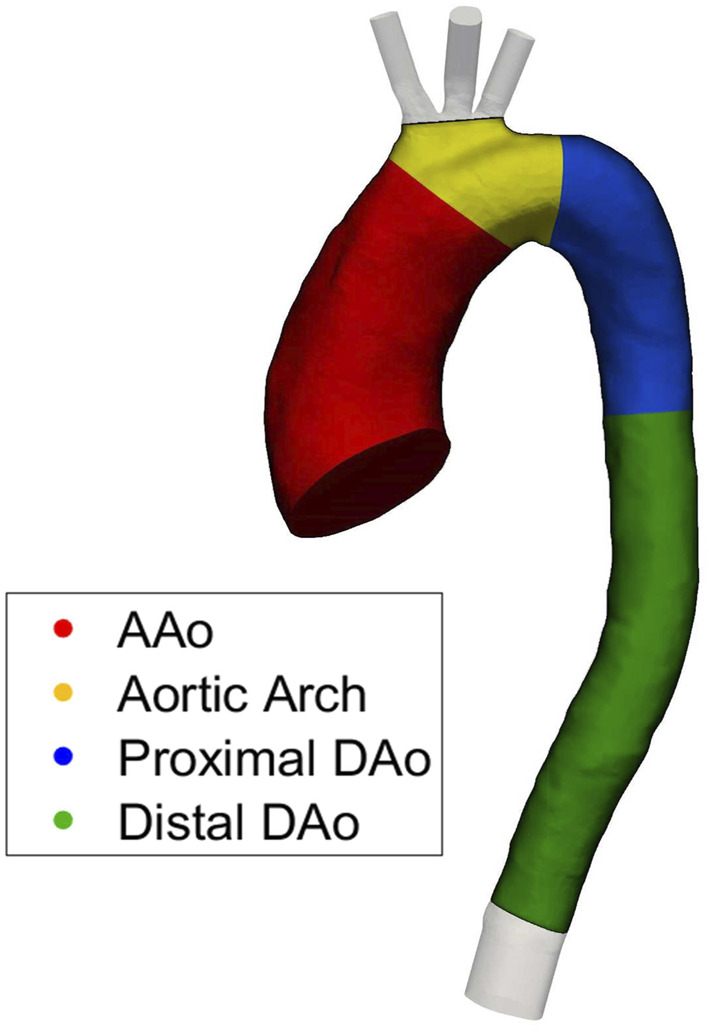
Four regions of interest (ROIs) used for quantitative regional comparisons.

To allow quantitative regional comparisons, the aorta was split into four regions of interest (ROIs) including the ascending aorta (AAo), aortic arch, proximal descending thoracic aorta (DAo), and distal DAo as shown in Figure 1. For each region, selected haemodynamic parameters are spatially integrated over each ROI, providing a spatial average.

In this study we used the LES simulation results as the baseline, meaning ILR-, LR- and HR-Laminar simulation results are compared directly to LES. Accuracy of the LES approach was previously evaluated in an idealised case (Manchester and Xu, 2020) and in the same aorta case used in this study (Manchester et al., 2021). The 4D flow MRI data acquired for this patient provides three-component velocities over the aortic volume, at 20 time points in a cardiac cycle. 4D flow MRI has limited spatiotemporal resolutions (compared to LES) which may compromise accuracy, especially for parameters derived from spatial gradients (e.g., wall shear stress) (Petersson et al., 2012). Turbulence statistics were not acquired with MRI, therefore it is only possible to make direct comparison of velocities between the computational results and 4D flow MRI measurement. All wall shear stress and turbulence-related measures are compared with those calculated from the LES results.

## 3 Results

### 3.1 Simulation Comparisons

Table 3 includes details on simulation lengths and time-step convergence. The LR-Laminar simulation took ∼1.5 days to complete 30 cardiac cycles and both the HR-Laminar and LES have comparable simulation times taking ∼10 days for 30 cardiac cycles. On average, 32 iterations per time-step were required to achieve convergence of pressure and velocity in the LR-Laminar simulation. In the HR-Laminar and LES simulations an average of 23 and 21 iterations per time-step were required to achieve convergence of pressure and velocity. Convergence was achieved at all time-steps for all simulation types. Further analysis showed that HR-Laminar required more iterations throughout systolic deceleration.

**TABLE 3:** Simulation times and convergence details.

Simulation Type	Cores	Simulation time [hours]	Percentage of converged time-steps	Average iterations per converged time-step
LR-Laminar	216	37.1	100.0%	32
HR-Laminar	216	240.9	100.0%	23
LES	216	244.3	100.0%	21

### 3.2 Comparison with 4D Flow Magnetic Resonance Imaging

The three simulated velocity fields are quantitatively compared to 4D flow MRI using the Pearson’s correlation method which gives a normalised measure of the covariance of two variables, quantifying the linearity between two data-sets Mukaka (2012). The Pearson’s product-moment correlation coefficient (R) for each velocity component is calculated over the entire aortic fluid domain, providing a point-by-point comparison. CFD velocity fields are down sampled to match 4D flow MRI resolution, as recommended in Puiseux et al. (2019). Values are given in Table 4 and correlation plots for the velocity components are provided in Supplementary Material. R > 0.7 indicate a high positive correlation and R > 0.5 indicate a moderate positive correlation (Mukaka, 2012). For each of the three velocity components, all simulations show a high positive correlation with 4D flow MRI velocities, except ILR-Laminar. ILR-Laminar post-processed with instantaneous velocities showed a high positive correlation in the x and y-components of velocity and a moderate positive correlation in the z-component of velocity. All simulations can accurately model velocities at peak systole–regardless of numerical or post-processing approach.

**TABLE 4:** Pearson correlation coefficients (R) for the three components of velocity, calculated using the entire aortic fluid domain. R is calculated using instantaneous velocities in ILR-Laminar, and phase-averaged velocities in LR-, HR-Laminar and LES.

Simulation Type	R component		
	*u*_*x*_	*u*_*y*_	*u*_*z*_
ILR-Laminar	0.76	0.78	0.69
LR-Laminar	0.82	0.81	0.78
HR-Laminar	0.81	0.82	0.76
LES	0.84	0.83	0.80

### 3.3 General Flow Features

**FIGURE 2:**
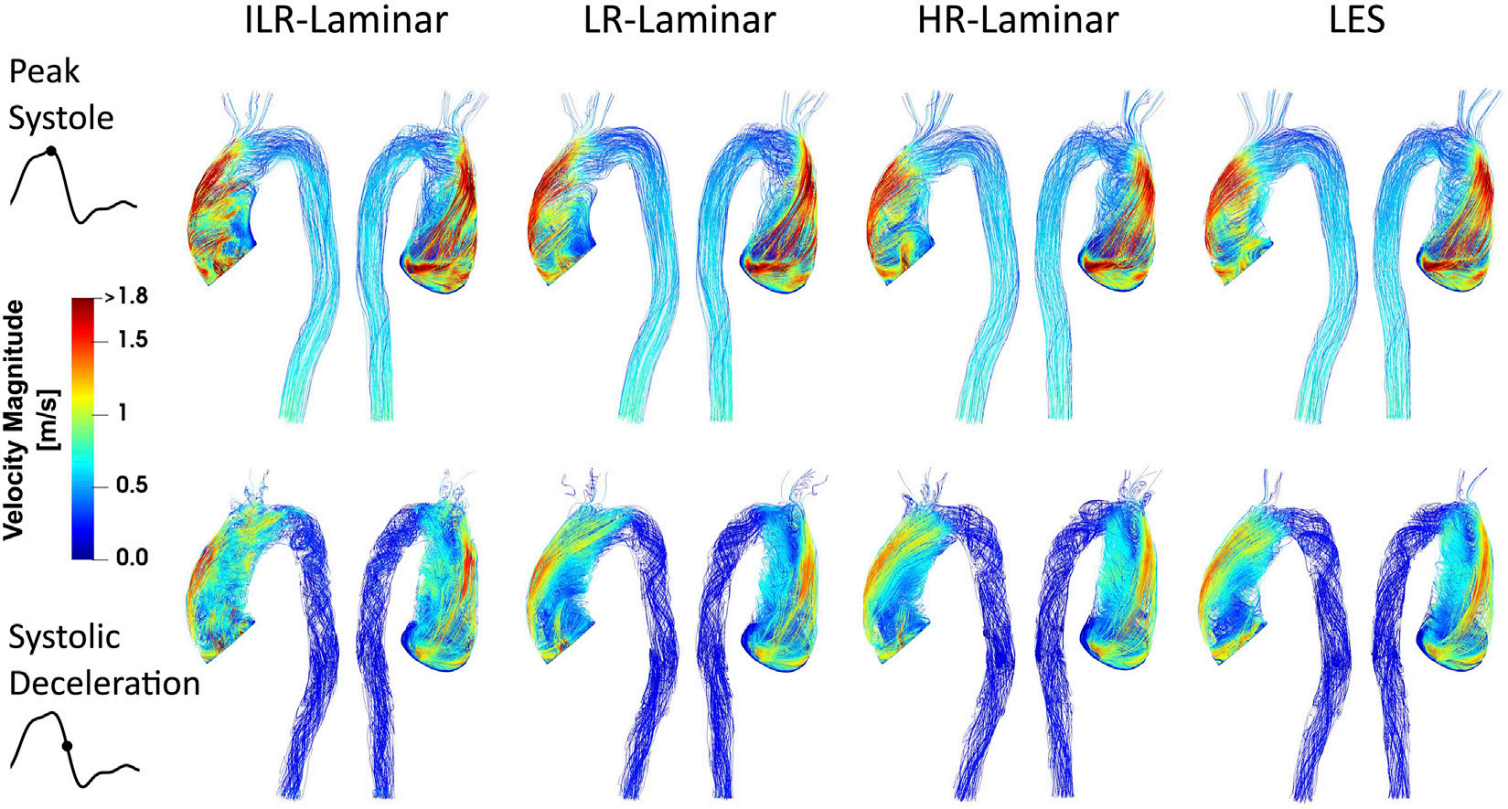
Velocity magnitude streamlines at peak systole (top row) and systolic deceleration (bottom row) for ILR-Laminar, LR-Laminar, HR-Laminar and LES simulations.

Velocity magnitude streamlines at two systolic time-points are visualised in Figure 2 for the three simulations. For LR-Laminar, both instantaneous and phase-averaged velocities are presented. For HR-Laminar and LES simulations, only phase-averaged velocities are shown. LR-Laminar and HR-Laminar velocity streamlines show good qualitative agreement with LES streamlines throughout the aorta, regardless of the post-processing approach (instantaneous or phase-average). Furthermore, there is good agreement at both peak systole and systolic deceleration. Primary flow features are well predicted, including the high velocity and skewed aortic valve flow which impinges on the anterior vessel wall. LR-Laminar instantaneous velocity streamlines (ILR-Laminar) are more chaotic, particularly in the deceleration phase.

### 3.4 Turbulence Kinetic Energy

**FIGURE 3:**
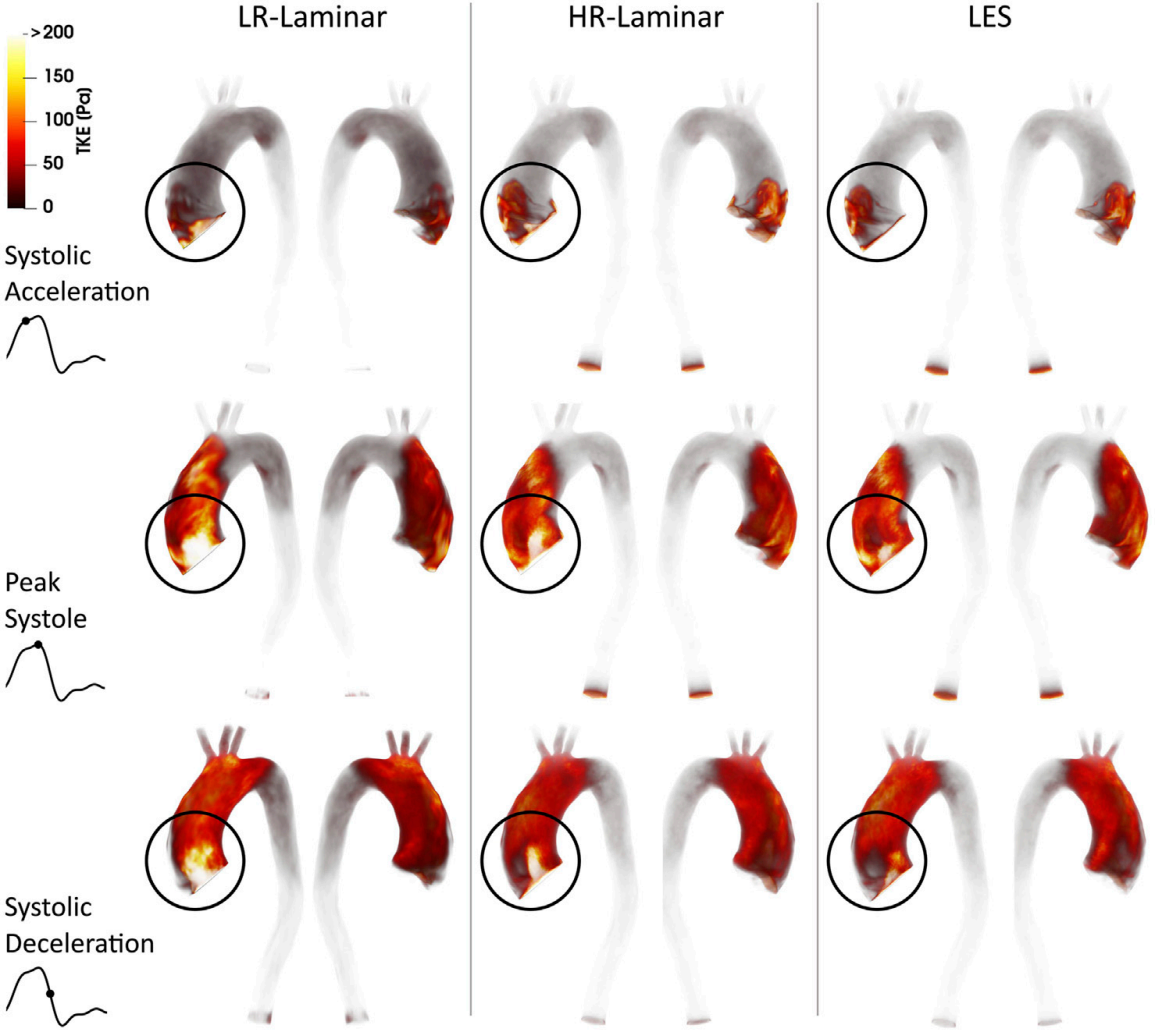
Turbulence kinetic energy (TKE) volume renderings for LR-Laminar, HR-Laminar and LES simulations at three time-points. Top-to-bottom: systolic acceleration, peak systole and systolic deceleration. Locations of largest differences are circled.

Volume renderings of turbulence kinetic energy (TKE) at three time-points in the cardiac cycle are shown in Figure 3. Turbulence production is primarily attributed to the stenosed aortic valve which produces a high velocity and skewed jet. This jet enters the lower velocity fluid in the dilated AAo and impacts on the arterial wall, with the dilated AAo providing space for turbulence to develop. Highest TKE values are found in the AAo and aortic arch, with smaller values in the descending thoracic aorta. Visually, TKE patterns are relatively well predicted by both LR- and HR-Laminar simulations, although TKE values are notably higher near the computational model inlet. These locations of largest differences are highlighted with circles in Figure 3.

**FIGURE 4:**
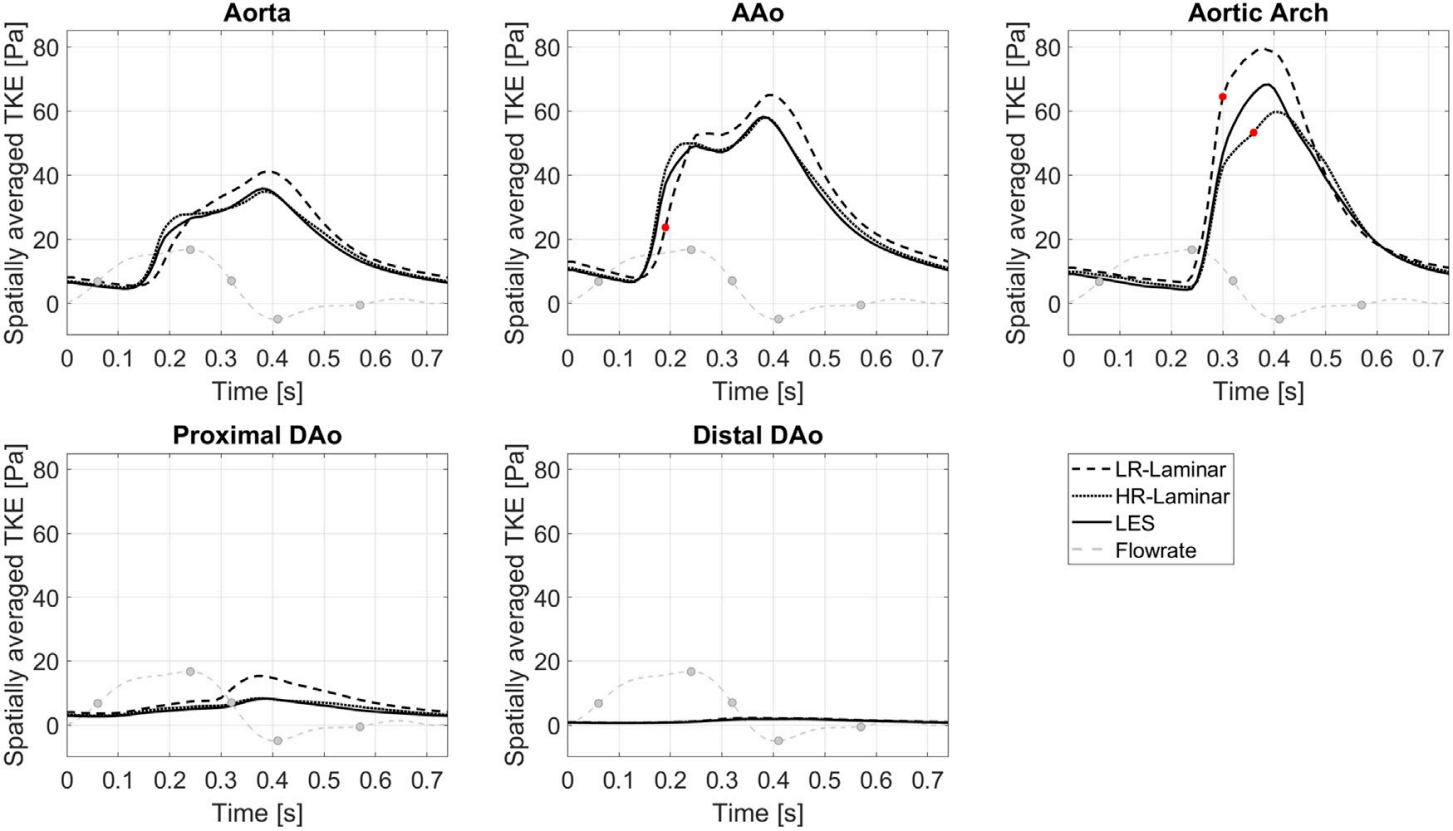
TKE spatially averaged over the entire aorta and each ROI, plotted over the cardiac cycle. Largest differences relative to LES are indicated by red markers. Key times throughout the cardiac cycle are indicated by grey markers and refer to maximum acceleration, peak systole, maximum deceleration, end systole and mid-diastole.

Figure 4 shows TKE spatially averaged over the entire aorta and each ROI, plotted over the entire cardiac cycle. Upon visual inspection all three simulations show similar trends over the cardiac cycle although values differ. Relative to LES, HR-Laminar predicts spatially averaged TKE values well, except in the AAo near peak systole and in the aortic arch during systolic deceleration. In the arch, HR-Laminar underpredicts spatially averaged TKE by up to 12.2 Pa (18.7% relative error). The LR-Laminar simulation typically overpredicts turbulence levels throughout the aorta, especially during systolic deceleration and diastole. In the AAo, spatially averaged TKE is underpredicted by 13.5 Pa (36.3% relative error) and in the arch, spatially averaged TKE is overpredicted by 17.9 Pa (38.4% relative error). Largest differences are indicated by red markers in Figure 4.

### 3.5 Wall Shear Stress

#### 3.5.1 Laminar Wall Shear Stress

**FIGURE 5:**
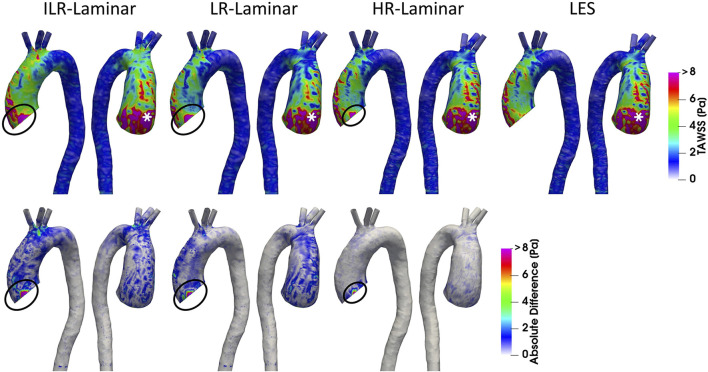
Top row: Time-averaged wall shear stress (TAWSS) contours for ILR-Laminar, LR-Laminar, HR-Laminar and LES simulations. Bottom row: Absolute difference in TAWSS values for ILR-, LR- and HR-Laminar simulations, relative to the LES simulation. Locations of interest are circled and asterisked.

Phase-averaged WSS is averaged over the cardiac cycle to give the time-averaged wall shear stress (TAWSS) for each of the three simulations. For the LR-Laminar simulation, the TAWSS is also calculated using instantaneous wall shear stresses from the last cardiac cycle. TAWSS contours are shown in Figure 5, alongside absolute differences in TAWSS between ILR-, LR- and HR-Laminar simulations and the LES simulation. Upon visual inspection, similar TAWSS patterns are seen in all simulations, regardless of post-processing approach. Largest differences occur along the left wall of the ascending aorta, near to the inlet (Figure 5, circled). This is likely an artefact of the inlet velocity contours which can artificially impose high near wall velocities. Excluding these regions (of potentially artificially high TAWSS), highest TAWSS’s occur in the ascending aorta along the anterior wall and are in excellent agreement, reaching peak TAWSS values of 14.9, 14.7, 15.0 and 14.9 Pa in the ILR-Laminar, LR-Laminar, HR-Laminar and LES simulations, respectively. Locations of peak TAWSS are denoted by the asterisks in Figure 5. Relative to LES, the ILR-, LR- and HR-Laminar peak TAWSS values correspond to absolute errors 0.3%, 1.4% and 0.5%, respectively.

**FIGURE 6:**
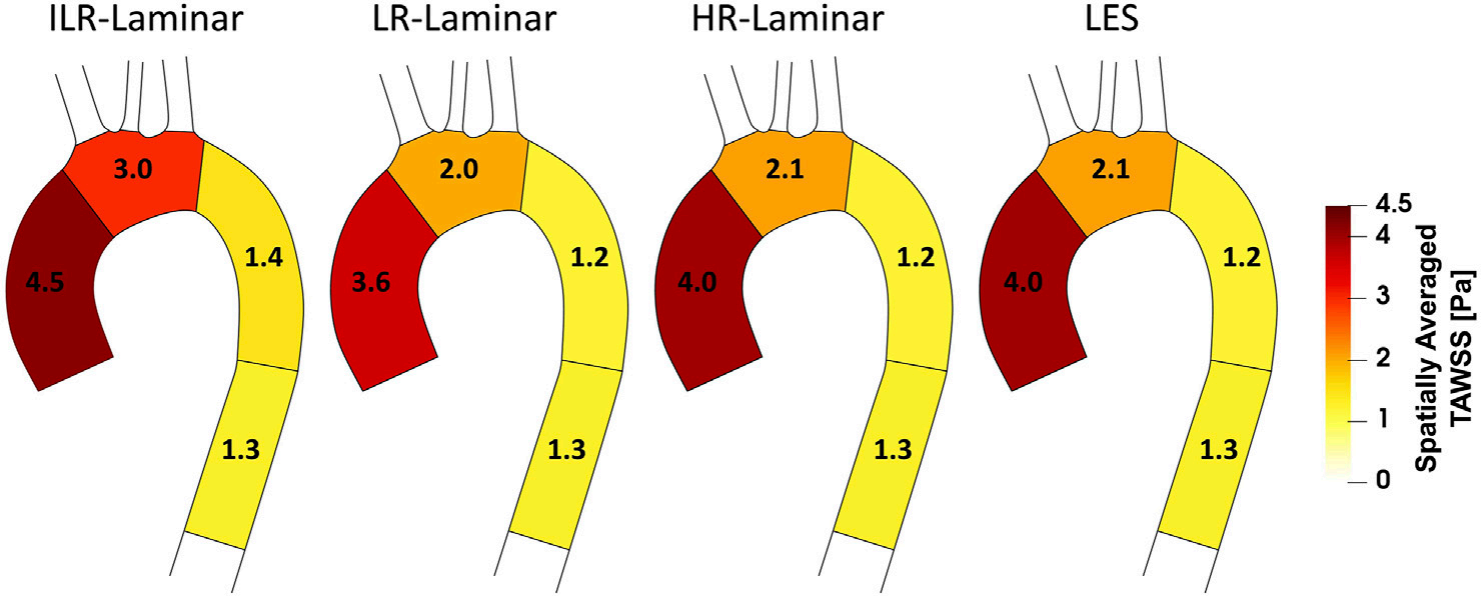
Regional analysis visualisation. Time-averaged wall shear stress (TAWSS) spatially averaged over regions of interest for ILR-Laminar, LR-Laminar, HR-Laminar and LES simulations.

Figure 6 shows a schematic of TAWSS spatially averaged over each ROI, for each simulation. Each ROI is colour-coded using the average value of TAWSS in that section. HR-Laminar and LES values are in excellent agreement in all ROIs showing identical values correct to 1 decimal place. In the LR-Laminar simulation, values are underpredicted in the AAo and arch. Largest differences up to 0.4 Pa are observed in the AAo (10.6% relative error to LES). In the ILR-Laminar case, values are overpredicted in the AAo, arch, and proximal DAo. Differences are less than 1 Pa in all ROIs, with largest differences observed in the aortic arch (44.6% relative error).

**FIGURE 7:**
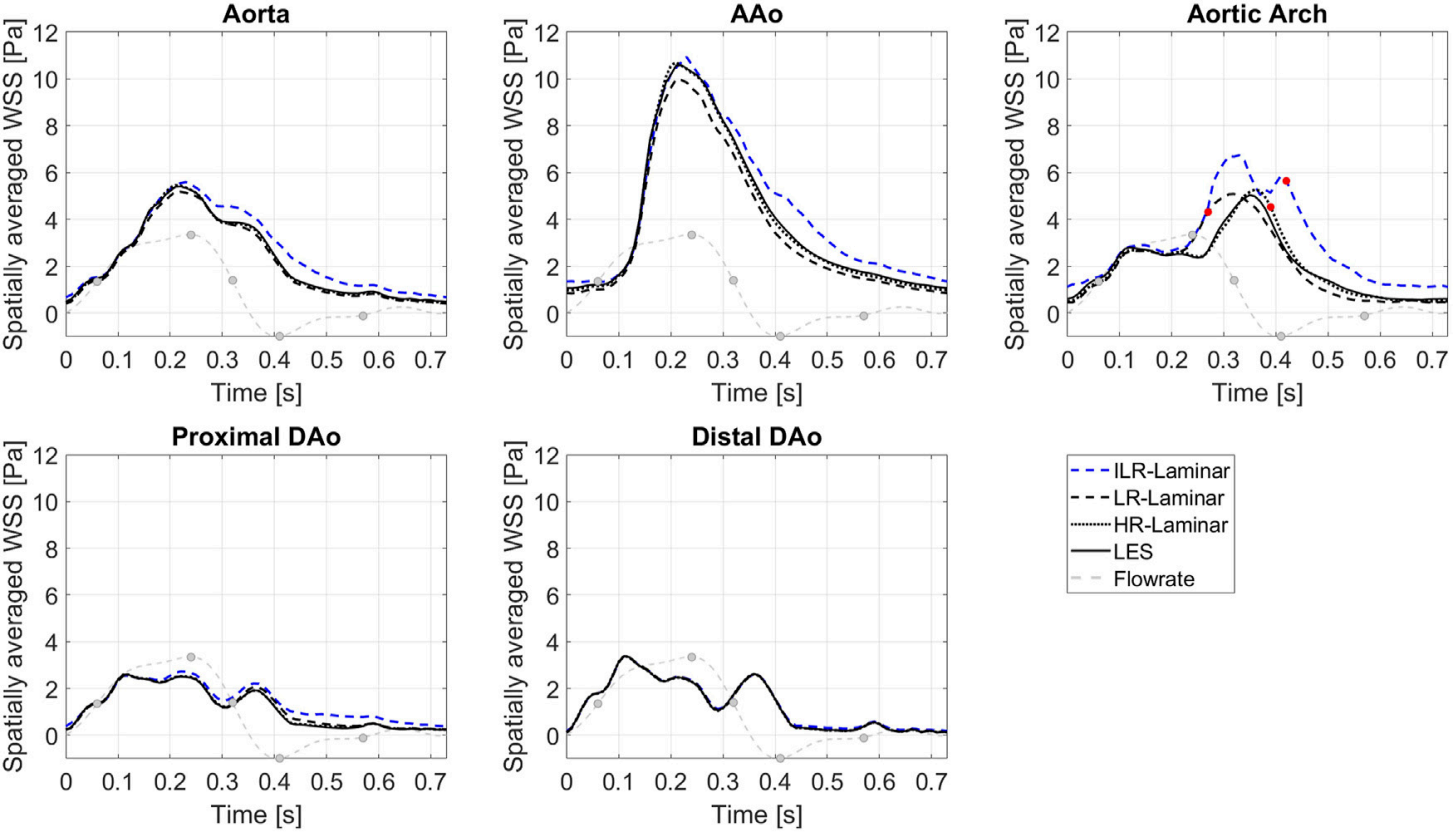
Wall shear stress (WSS) spatially averaged over the entire aorta and each ROI, plotted over the cardiac cycle. Largest differences relative to LES are indicated by red markers. Key times throughout the cardiac cycle are highlighted and refer to maximum acceleration, peak systole, maximum deceleration, end systole and mid-diastole.

Figure 7 shows WSS spatially averaged over the entire aorta and each ROI, plotted over the entire cardiac cycle. ILR-Laminar results are based on instantaneous WSS from the final cardiac cycle and LR-, HR-Laminar and LES results are based on phase-averaged WSS. Compared to the LES simulation, HR-Laminar shows excellent agreement over the cardiac cycle in all regions. All differences are less than 0.6 Pa, with largest differences seen in the aortic arch near end systole. Both ILR- and LR-Laminar simulation results capture similar WSS trends over the cardiac cycle in all regions. Good agreement is seen in the proximal and distal DAo regions (differences less than 0.6 Pa in both simulations), with larger differences seen in the AAo and aortic arch. For ILR-Laminar, largest differences of 3.0 Pa occur in the aortic arch near end systole, and in LR-Laminar, largest differences of 1.7 Pa occur in the arch near peak systole. Largest differences are indicated by red markers in Figure 7.

#### 3.5.2 Turbulent Wall Shear Stress

**FIGURE 8:**
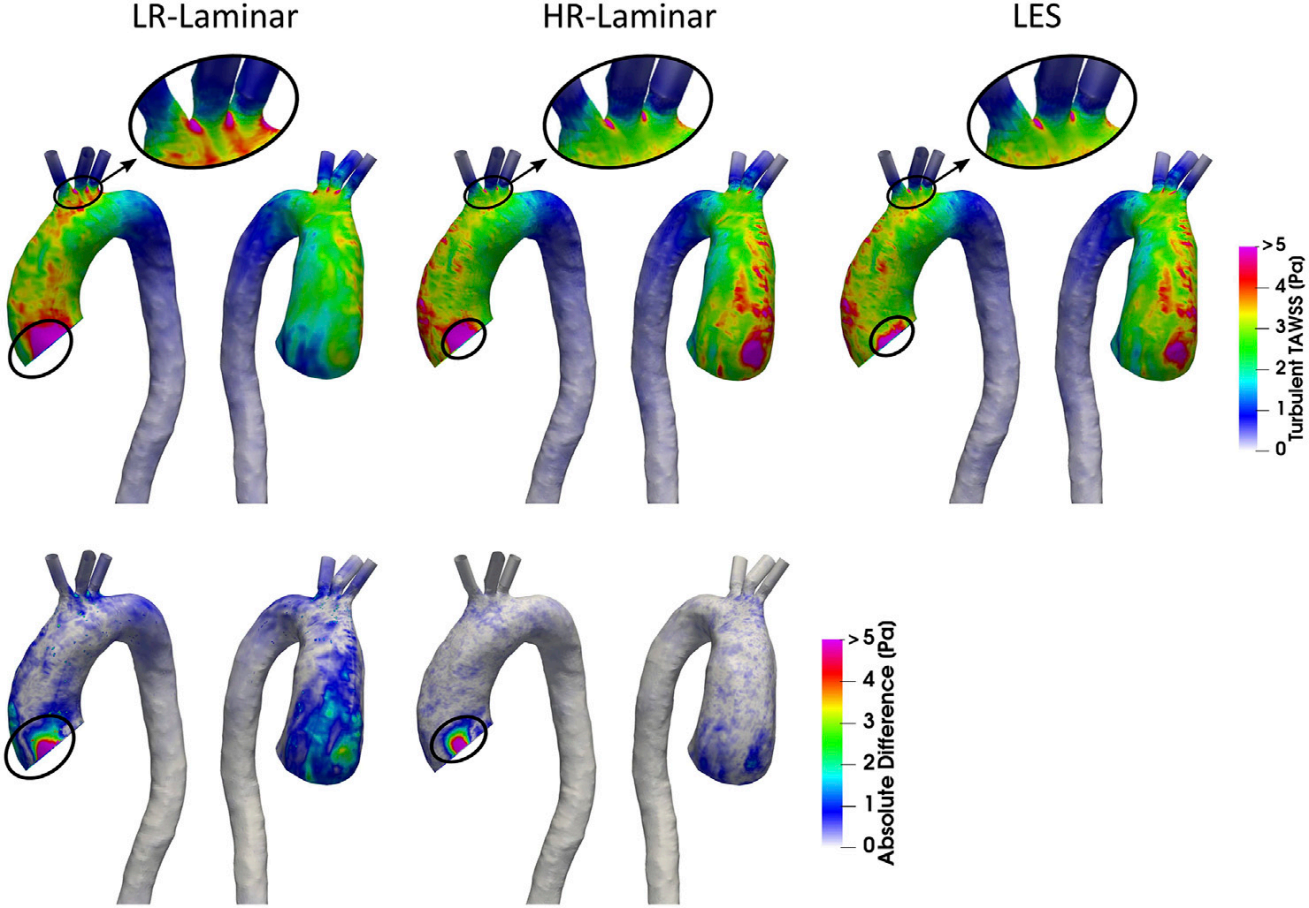
Top row: Time-averaged turbulent wall shear stress (Turbulent-TAWSS) contours for LR-Laminar, HR-Laminar and LES simulations. Bottom row: Absolute difference in turbulent-TAWSS values for LR- and HR-Laminar simulations, relative to the LES simulation. Locations of interest are circled.

Turbulent phase-averaged WSS is averaged over the cardiac cycle to give the time-averaged turbulent wall shear stress (turbulent-TAWSS) for LR-, HR-Laminar and LES simulations. Turbulent-TAWSS contours are shown in Figure 8, alongside absolute differences in turbulent-TAWSS between LR-, HR-Laminar simulations and the LES simulation. Visually, turbulent-TAWSS patterns agree well over the aorta, except near the inlet (Figure 8, circled). Excluding peak values near the inlet, highest turbulent-TAWSS’s are experienced between the aortic arch branches in all simulations (Figure 8, circled). At these locations, the LR-Laminar, HR-Laminar and LES simulations each show peak values of 14.7, 11.5 and 11.2 Pa, respectively. Relative to LES, the LR- and HR-Laminar peak turbulent-TAWSS values correspond to maximum absolute differences of 3.5 Pa and 0.3 Pa (absolute relative errors of 31.3% and 2.7%).

**FIGURE 9:**
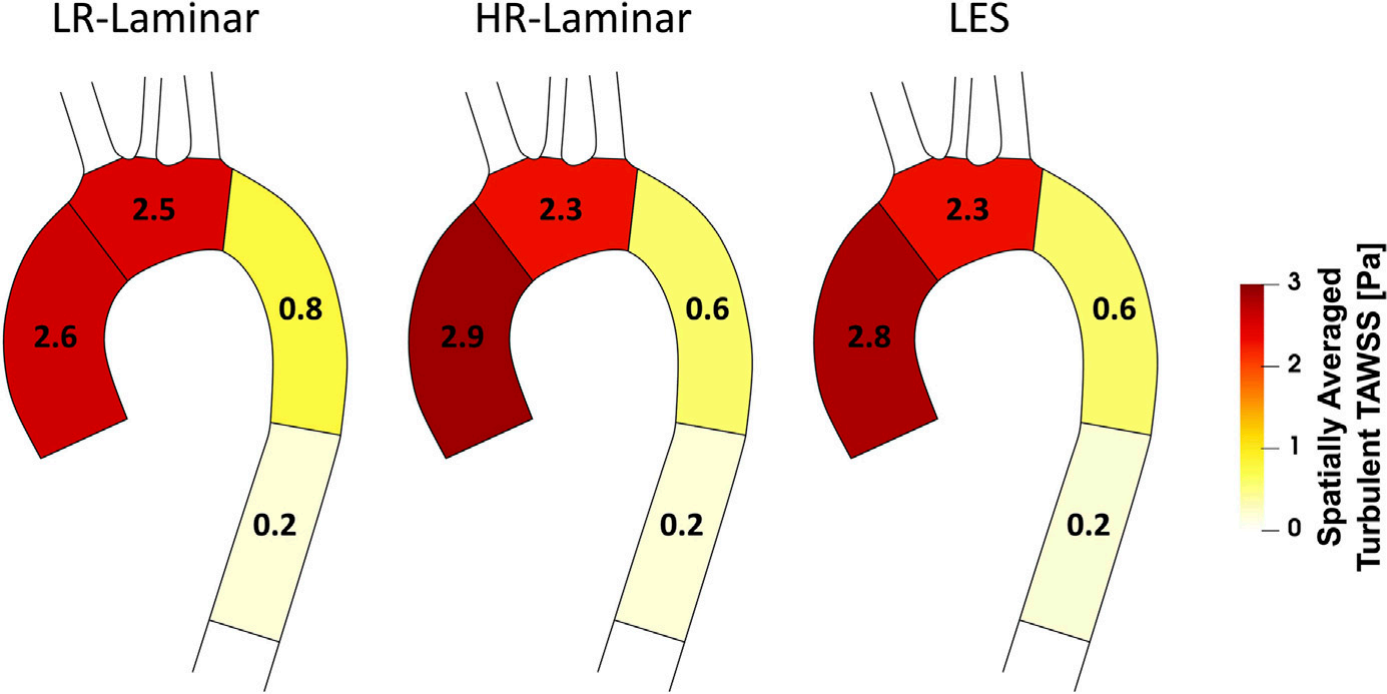
Regional analysis visualisation. Time-averaged turbulent wall shear stress (Turbulent-TAWSS) spatially averaged over regions of interest for LR-Laminar, HR-Laminar and LES simulations.

A schematic of turbulent-TAWSS spatially averaged over each ROI is shown in Figure 9 for each simulation. HR-Laminar and LES predicted values are in good agreement with differences < 0.2 Pa in all ROIs (5.1% relative error in the AAo). The LR-Laminar simulation underpredicts turbulent-TAWSS in the AAo and overpredicts turbulent-TAWSS in the aortic arch and proximal descending thoracic aorta. Relative to LES, differences are less than 0.3 Pa with largest differences observed in the proximal descending thoracic aorta (38.6% relative error).

**FIGURE 10:**
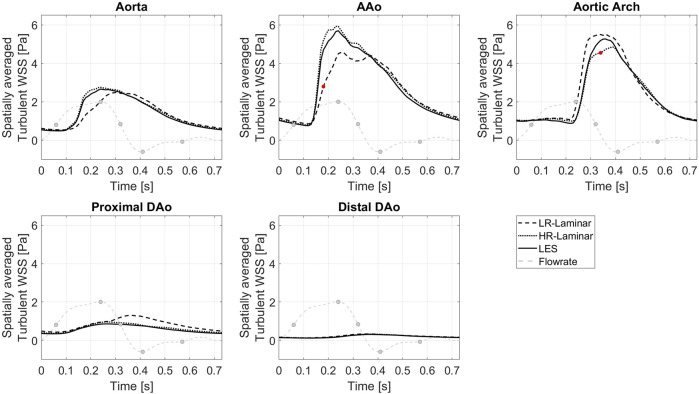
Turbulent WSS spatially averaged over the entire aorta and each ROI, plotted over a cardiac cycle. Largest differences relative to LES are indicated by red markers. Key times throughout the cardiac cycle refer to maximum acceleration, peak systole, maximum deceleration, end systole and mid-diastole.

Figure 10 shows the turbulent-WSS spatially averaged over the entire aorta and each ROI, plotted over the entire cardiac cycle. Compared to the LES simulation, HR-Laminar shows similar turbulent-WSS behaviours over the cardiac cycle in all regions except in the aortic arch, with differences up to 0.7 Pa during systolic deceleration. LR-Laminar turbulent-WSS trends differ to LES with maximum differences reaching 1.9 Pa in the AAo before peak systole. Largest differences are shown by red markers in Figure 10.

#### 3.5.3 Oscillatory Shear Index

**FIGURE 11:**
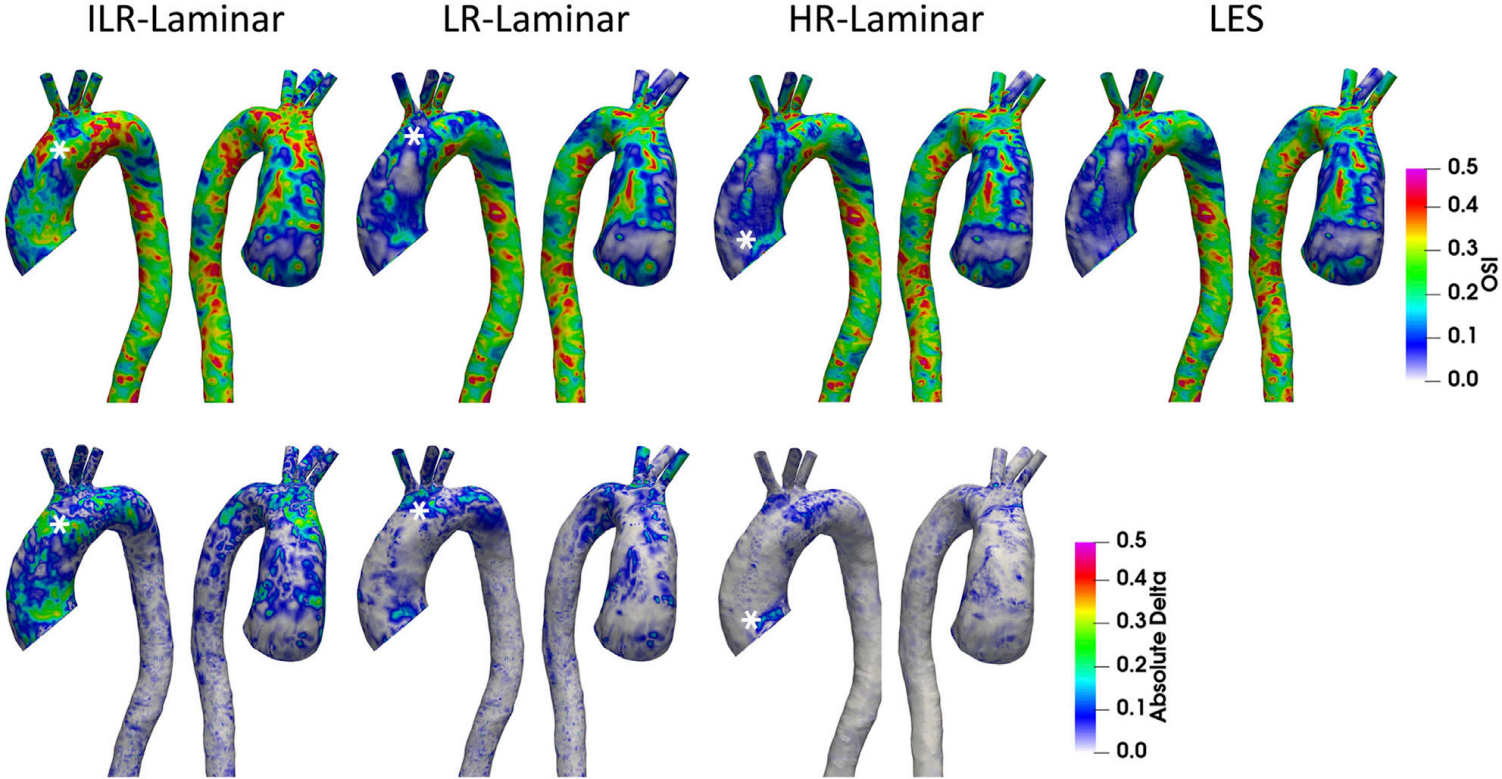
Top row: Oscillatory shear index (OSI) contours for ILR-Laminar, LR-Laminar, HR-Laminar and LES simulations. Bottom row: Absolute difference in OSI values for ILR-, LR- and HR-Laminar simulations, relative to the LES simulation. Locations of largest differences are indicated with asterisks.

OSI is a dimensionless measure of WSS alignment and quantifies deviation of the WSS vector from the TAWSS vector over the cardiac cycle. A value of 0 indicates complete alignment throughout the cardiac cycle and a value of 0.5 indicates the converse. OSI contours are shown in Figure 11 alongside differences in OSI between ILR-, LR- and HR-Laminar simulations and the LES simulation. OSI contours are visually similar, and best agreement is seen in HR-Laminar with differences up to 0.23. ILR- and LR-Laminar OSI both showed larger differences up to 0.48, relative to the LES simulation. This means that in certain regions ILR- and LR-Laminar simulations show opposite OSI results to the LES simulation. Locations of largest differences are indicated with an asterisk in Figure 11.

### 3.6 Energy Loss

#### 3.6.1 Laminar Viscous Energy Loss

**FIGURE 12:**
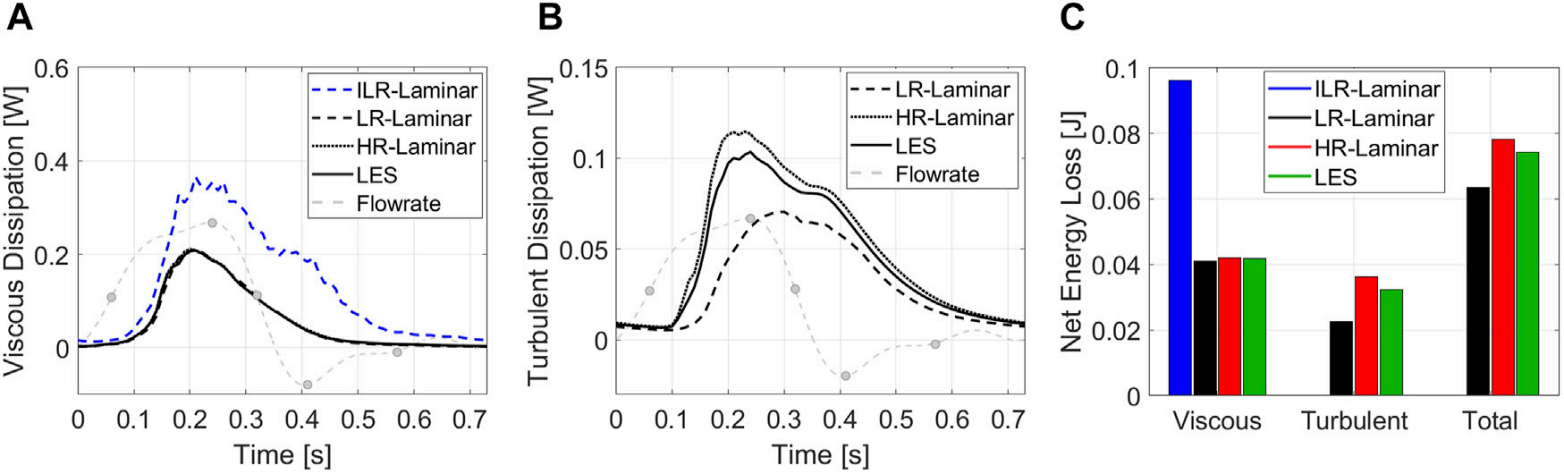
**(A)** Viscous dissipation and **(B)** turbulent dissipation spatially averaged over the entire aorta, plotted over the cardiac cycle. **(C)** Net energy losses. Key times throughout the cardiac cycle are highlighted and refer to maximum acceleration, peak systole, maximum deceleration, end systole and mid-diastole.

Viscous dissipation over the cardiac cycle is plotted in Figure 12A, for all three simulations. LR-, HR-Laminar and LES simulations show similar behaviours over the cardiac cycle, all peaking just ahead of peak systole. ILR-Laminar shows similar trends although values are massively overpredicted. Relative to the LES simulation, ILR-, LR- and HR-Laminar show errors of 75.6%, 1.7% and 1.5% in peak viscous dissipation values, respectively. The net viscous energy loss is calculated by integrating the viscous dissipation over the cardiac cycle and is shown in Figure 12C. ILR-, LR- and HR-Laminar show 130.3%, 1.7% and 0.8% errors relative to the LES simulation.

#### 3.6.2 Turbulent Viscous Energy Loss

Turbulent dissipation over the cardiac cycle is plotted in Figure 12B, for all three simulations. HR-Laminar and LES simulations show similar behaviours over the cardiac cycle, although values differ. Relative to the LES simulation, LR-Laminar show largest differences near peak systole with 54.4% relative error. HR-Laminar also has largest differences near peak systole of 22.8% relative error. The net turbulent energy loss is calculated by integrating the turbulent dissipation over the cardiac cycle and is shown in Figure 12C. LR- and HR-Laminar show 30.4% underprediction and 11.6% overprediction, respectively, relative to the LES simulation.

## 4 Discussion

Since the early conception of the Womersley flow model in the 1950s (Womersley, 1954; Hale et al., 1955a; Hale et al., 1955b; Womersley, 1955), blood flow in large arteries was assumed laminar and has typically been treated as such in numerical simulations. Recently, there has been a shift in attitudes towards the flow state of cardiovascular flows. In the past decade numerical studies accounting for blood flow disturbances are on the rise, finding turbulence features in both the pathologic and healthy aorta (Lantz et al., 2012; Lantz et al., 2013; Miyazaki et al., 2017; Xu et al., 2018; Saqr et al., 2020; Xu et al., 2020; Manchester et al., 2021). One such study conducted semi-patient-specific simulations of a healthy adult aorta and a child aorta with double aortic arch (Miyazaki et al., 2017). Three modelling approaches were used: laminar, LES and the renormalisation group (RNG) *k* − *ϵ* model. Similar to this study, velocities were quantitatively compared to 4D flow MRI velocities using the Pearson’s correlation method. They found that predicted velocities from the RNG *k* − *ϵ* model correlated marginally better than laminar and LES velocities, although poorer correlations (low to moderate) were observed in the child AAo owing to the flat inlet velocity profile which lacks secondary velocities. WSS values and laminar viscous energy losses from all three simulations did not correlate well with values calculated directly from 4D flow MRI because of lower spatial resolution. It was unclear which modelling approach performed the best in their study.

### 4.1 ILR-, LR- and HR-Laminar Comparisons with LES

In the present study, peak systolic velocities compared very well to 4D flow MRI velocities, throughout the entire aortic fluid domain. All simulations achieved a high positive correlation, except the z-component in the ILR-Laminar simulation which was just below the threshold and indicated a moderate positive correlation. Similarly, velocity streamlines were well predicted by all simulations, even during systolic deceleration when the flow-state is highly unstable. For this patient, the results suggest that any of the simulation types, including ILR-Laminar, could be used to predict velocities and flow patterns to a reasonable degree of accuracy. Visually, primary turbulence features are captured by all three simulations and spatial-temporal trends were similar. Both LR- and HR-Laminar simulations overestimated turbulence production near the inlet. Quantitatively, HR-Laminar TKE compared well to LES TKE throughout the cardiac cycle except in the aortic arch during systolic deceleration, and LR-Laminar typically overpredicted TKE over the cardiac cycle.

In terms of wall shear stresses and OSI, HR-Laminar compared best with LES predicted values. For phase-averaged (laminar) wall shear stress; maximum TAWSS was predicted within 0.5% relative accuracy, ROI analysis showed that TAWSS agreed in all regions to one decimal place, and WSS plotted over the cardiac cycle showed differences less than 0.6 Pa. In general, excellent agreement was observed between HR-Laminar and LES mean wall shear stresses. Larger differences were observed in turbulent wall shear stresses; maximum turbulent-TAWSS differed by 2.7%, ROI analysis showed that turbulent-TAWSS was overpredicted in the AAo (5.1% relative error) but was correct elsewhere, and regional-temporal analysis of turbulent-WSS showed differences less than 0.7 Pa. Overall, turbulent wall shear stresses are typically well predicted by the HR-Laminar simulation, but differences in values were observed. OSI was also fairly well predicted by HR-Laminar, although differences up to 0.23 were seen.

ILR-Laminar and LR-Laminar wall shear stresses and OSI did not compare so favourably to LES. In terms of phase-averaged (laminar) wall shear stress the lower resolution simulations were able to capture trends–both TAWSS contours and WSS plotted over the cardiac cycle are visually similar to LES—but quantitative analysis showed that values differ significantly. ILR- and LR-Laminar simulations showed relative errors of 0.3% and 1.4% in peak TAWSS values. ROI analysis showed that both simulations predicted TAWSS relatively well in the DAo, but large differences were seen in the AAo of LR-Laminar (10.6% error) and in the aortic arch of ILR-Laminar (44.6% error). Temporal and spatial analysis showed that WSS and TAWSS were better predicted in regions of laminar or lowly disturbed flow (Figure 7). E.g., in the proximal and distal DAo where TKE is small throughout the cardiac cycle and in the AAo and aortic arch during systolic acceleration when TKE is small. In the AAo and aortic arch during systolic deceleration and diastole, TKE levels are high and/or dissipating, and the low-resolution simulations cannot accurately predict WSS and TAWSS. These findings agree well with those of Xu et al. (2020) who compared laminar and LES simulations of three patient-specific aortas with dilation and different aortic valve morphologies. They found little difference in large-scale flow parameters, with laminar simulations underpredicting TAWSS by up to 5%. The authors observed largest differences in localised regions of highly disturbed flow—particularly in the aorta with severe aortic valve stenosis. For turbulent wall shear stresses, LR-Laminar could not accurately estimate turbulent-WSS values and typically overpredicted values, consistent with TKE overpredictions. Peak turbulent-TAWSS differed by 31.3% and ROI analysis showed differences in all regions up to 38.6%. Considering LR-Laminar could not accurately predict WSS values, it is not surprising that the spatial and temporal resolution of the simulation was not sufficient in predicting turbulent-WSS as well. OSI contours were visually similar to LES, but accuracy diminished with simulation resolution which is also not unexpected considering OSI is based on WSS.

LR- and HR-Laminar viscous dissipations and energy losses were comparable to LES with viscous energy loss values up to 1.7% relative error. ILR-Laminar viscous energy losses showed the largest relative errors of all parameters included in this study of 130.3% and viscous dissipation was overpredicted over the entire cardiac cycle. Because the instantaneous velocity field is used in the calculation which is based on the velocity gradient tensor, fluctuations are not damped and are amplified when calculating the gradient. LES turbulent dissipation values proved challenging to match with LR- and HR-Laminar simulations, although trends over the cardiac cycle were comparable in HR-Laminar. LR-Laminar underpredicted turbulent energy losses by 31.4% and HR-Laminar overpredicted it by 11.6%.

Comparing HR-Laminar and LES simulation results, it is clear that the contribution from the subgrid-scale model (or lack of) has a notable influence on predicted turbulence-based results in this case.

### 4.2 Post-Processing Approaches

In typical laminar-based simulations of the aorta, simulations are run until certain parameters are deemed to have reached a periodic solution. Pressure at the branch outlets is monitored, and once this pressure has reached a periodic solution, it is assumed that all other properties have also reached a periodic solution and the simulation is stopped. Following this, results obtained in the prior cardiac cycles are neglected, and post-processing is conducted on the final cycle only using instantaneous parameters. The results from this study show that although a periodic solution in pressure is easily achieved (at 8 cardiac cycles), it does not necessarily imply that a periodic solution in all parameters is achieved and that there are still cycle-to-cycle variations. This is revealed by comparing the results from ILR- and LR-Laminar simulations, where the same simulation results were post-processed using two different approaches. Only laminar-based parameters were compared because turbulence-based parameters cannot directly be calculated from instantaneous values. There was little difference in the output velocities between the two post-processing approaches, but wall shear stresses were different. For this case, wall shear stress estimations were much better with the phase-averaged approach, although there were still deviations from the HR-Laminar and LES results. ILR-Laminar viscous energy losses were massively overpredicted, but LR-Laminar energy losses were in better agreement with LES.

### 4.3 Limitations, Future Work and Recommendations

In this study, the aortic wall was assumed rigid and valve leaflet motion was not directly modelled although effects were accounted for by making use of 4D flow MRI data. Whilst aortic wall motion may affect simulation results (Tan et al., 2009), the LES methods used in this paper have been thoroughly sensitivity tested and validated in idealised and patient-specific cases (Manchester and Xu, 2020; Manchester et al., 2021). Blood flow was treated as Newtonian which is widely considered an acceptable simplification in computational modelling of aortic flows. Real blood is Non-Newtonian and the length scales of red blood cells are not much smaller than the expected smallest length scales of blood flow turbulence (Antiga and Steinman, 2009). It is therefore reasonable to expect additional turbulence damping to occur at the smallest turbulence scales in Non-Newtonian flow. Andersson et al. (2015) found slight turbulence damping effects in an aortic coarctation model although this had little impact on general flow characteristics. Other studies into arterial flows found that a Newtonian flow assumption produced reasonably accurate results and that haemodynamic parameters were far more sensitive to geometric variability (Lee and Steinman, 2007; Marrero et al., 2014). Nonetheless, future studies could evaluate turbulence characteristic sensitivity to Newtonian and Non-Newtonian modelling approaches, as well as evaluate interactions with current subgrid-scale models which are designed to satisfy the properties of fully turbulent flows. In this study, an aortic case with severe aortic valve stenosis was selected to evaluate the various laminar-type simulations. Because this case showed high turbulence levels in a former study (Manchester et al., 2021), it was expected to provide a challenging test case for laminar-type simulations. Based on our findings, it is reasonable to hypothesise that laminar-type simulations of aortic flows with healthy valve types and less severe valve stenosis would perform better than the case considered in this study because turbulence levels are expected to be of smaller magnitude. Nonetheless, this study is limited to a single aortic case and in future work, a selection of aortas with a range of diseases and disturbance levels should be included to improve best practice surrounding the appropriate selection of computational approach. Only then can the results be generalised to all aortic flows.

Based on the findings from this paper, it is recommended that future numerical studies on aortic flows select the modelling approach based not only on expected flow state but the parameters of interest. For example, if only velocities are required then an ILR-Laminar type simulation may be appropriate. Considering LES simulations are computationally demanding and produce large amounts of data, an LES approach is not always feasible (e.g., in large scale studies) and alternative modelling approaches must be considered. HR-Laminar simulation results were less accurate than LES and simulation times were almost identical. Based on this, there was no benefit to running a higher resolution laminar simulation over LES. Comparing ILR-Laminar and LR-Laminar results showed that phase-averaging improved wall shear stress and viscous energy loss estimations in the lower resolution simulations. Adopting a more advanced post-processing approach is a relatively simple and low-cost way to improve simulation predictions.

## 5 Conclusion

Blood flow in a patient-specific aorta with aortic valve stenosis was simulated using different modelling approaches to assess their capabilities in capturing mean and turbulence-based parameters. Three modelling approaches were examined: LES, high-resolution (HR) laminar and low-resolution (LR) laminar. The HR-Laminar simulation used the same mesh and time-step as the LES simulation and is essentially a coarse DNS. The LR-Laminar simulation used a coarser mesh and larger time-step representative of typical laminar aortic simulations. Two post-processing approaches were compared using the LR-Laminar simulation results: one was based on the final periodic solution without phase-averaging (ILR-Laminar), and another involved phase-averaging of the same set of results over multiple cycles (LR-Laminar). A range of laminar and turbulence-based parameters were assessed.

All simulations, regardless of post-processing approach, could accurately predict velocities and flow patterns throughout the aorta. Lower resolution simulations (ILR- and LR-Laminar) were incapable of accurately predicting other laminar-based parameters calculated from velocity gradients (wall shear stress and viscous energy loss), although adopting a phase-averaged post-processing approach improved predictions. The higher resolution simulation (HR-Laminar) produced more comparable results to LES and laminar-based parameters were better estimated than turbulence-based parameters. The findings from this study suggest that well-resolved laminar simulations (HR-Laminar) may provide accurate estimations of laminar-based parameters in disturbed flows, although LES and HR-Laminar simulation times were identical; negating the benefits of running a laminar-type simulation over LES. Post-processing simulation results with a phase-averaged approach is a simple and low-cost way to improve accuracy of lower-resolution simulation results.

## Data Availability

The raw data supporting the conclusion of this article will be made available by the authors, without undue reservation.

## Nomenclature

AAo: ascending aorta
CFD: computational fluid dynamics
DAo: descending thoracic aorta
DNS: direct numerical simulation
HR: high resolution
ILR: instantaneous low resolution
LES: large-eddy simulation
LR: low resolution
MRI: magnetic resonance imaging
OSI: oscillatory shear index
RNG: renormalisation group
ROI: region of interest
SGS: subgrid-scale
TAWSS: time-average wall shear stress
TKE: turbulence kinetic energy
WALE: wall-adapting local eddy-viscosity
WSS: wall shear stress
