# Supplementary material for: Evaluation of Computational Methodologies for Accurate Prediction of Wall Shear Stress and Turbulence Parameters in a Patient-Specific Aorta
Source: Front Bioeng Biotechnol. 2022 Mar 24;10:836611. doi: 10.3389/fbioe.2022.836611 (PMC8987126; doi:10.3389/fbioe.2022.836611)
Supplement: Supplementary file 1 [file DataSheet1.pdf]

Supplementary Material

1 SPATIAL RESOLUTION

The resolution of the largest turbulence scales can be evaluated using two-point correlations (Davidson, 2009) and this approach was previously used to evaluate spatial resolutions in a patient-specific aortic coarctation (Andersson et al., 2015). The method estimates the number of mesh cells which resolve the largest spatial scales at a specified location. The normalised two-point correlation is given by:

$$B_{ij}(\boldsymbol{x}, t; \boldsymbol{r}) = \frac{\langle u'_i(\boldsymbol{x}, t) u'_j(\boldsymbol{x} + \boldsymbol{r}, t) \rangle}{u'_{i,RMS}(\boldsymbol{x}, t) u'_{j,RMS}(\boldsymbol{x}, t)} \tag{S1}$$

where RMS indicates root-mean-square. The probe was placed at a location in the ascending aorta with the highest turbulence levels (Figure S1A). Three-components of velocity were acquired at 20 point locations in the positive streamwise direction and 20 locations in the negative streamwise direction (blue arrow in Figure S1A). Similarly, three-components of velocity were also acquired in two positive radial directions and two negative radial directions (orange arrows in Figure S1A). This resulted in a total of 40 streamwise point acquisitions and 80 radial point acquisitions. In complex aortic flows, velocity components do not strictly follow a set direction (e.g., streamwise or radial). Therefore, data in each of the directions were averaged, providing an average correlation in the streamwise and radial direction, as shown in Figure S1B-C. A significant correlation is indicated by $B_{ij} > 0.2$. In the streamwise direction, all 20 cells were above this threshold, indicating very good resolution. In the radial direction, 8 cells are correlated with decorrelation ($B_{ij} < 0.1$) occurring after 12 cells. These results indicate sufficient resolution in both streamwise and radial directions.

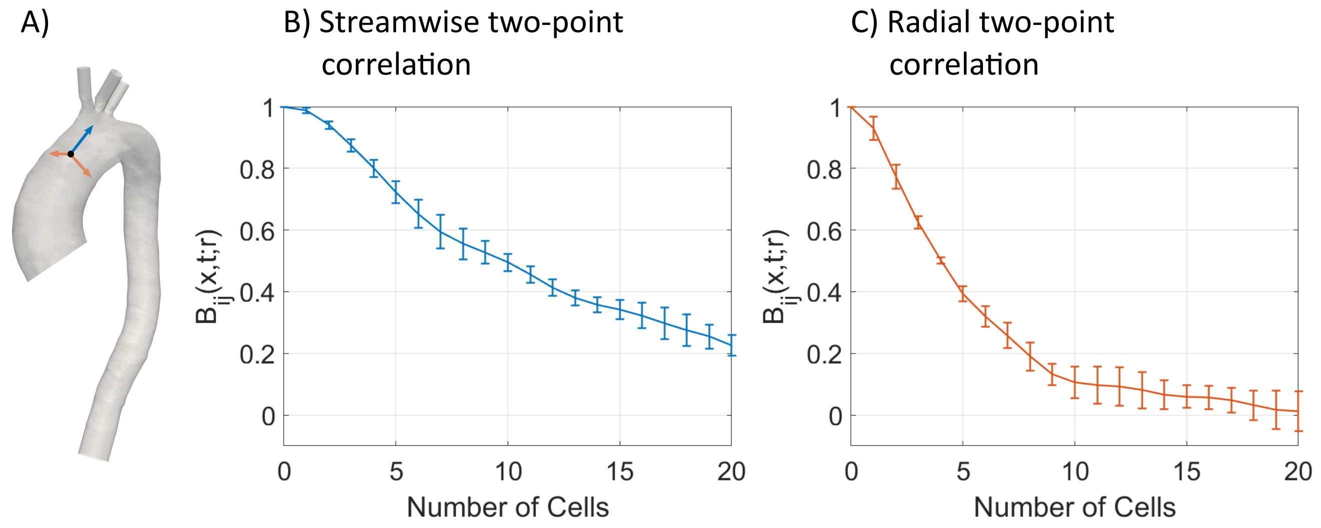

Figure S1. Average two-point correlations in the streamwise (B) and radial (C) directions with standard deviations. Data was obtained at a location in the ascending aorta with highest turbulence levels (A). The streamwise direction is indicated by the blue arrow and the two radial directions are indicated by the orange arrows (A).

2 SUPPLEMENTARY FIGURES

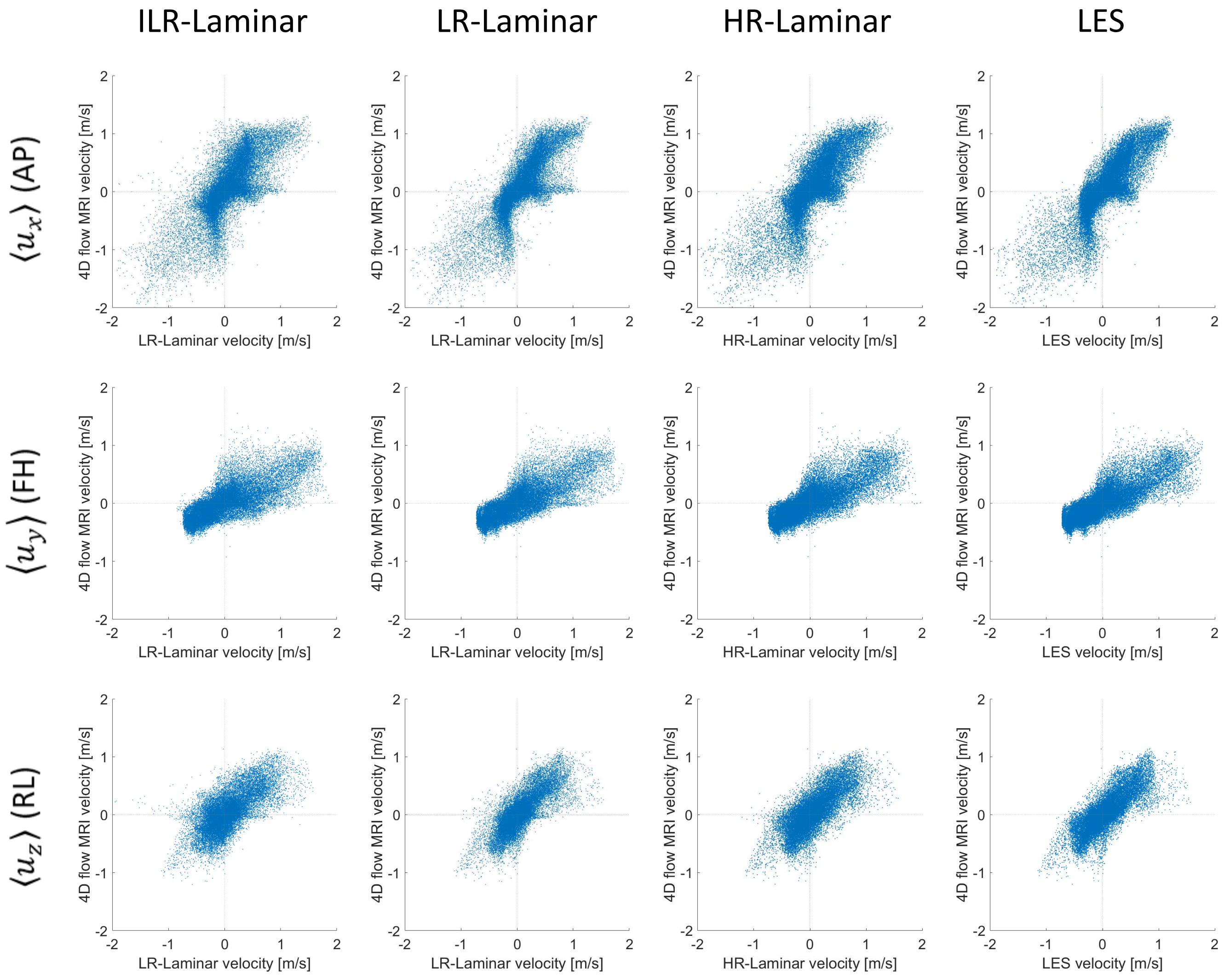

Figure S2. Correlation plots between 4D flow MRI and CFD simulations in the aorta at peak systole. Left-to-right: ILR-Laminar, LR-Laminar, HR-Laminar and LES. Top-to-bottom: Velocity components corresponding to anterior-posterior (AP), foot-head (FH) and right-left (RL) which correspond to x, y, z co-ordinates respectively.

REFERENCES

Andersson, M., Lantz, J., Ebbers, T., and Karlsson, M. (2015). Quantitative assessment of turbulence and flow eccentricity in an aortic coarctation: Impact of virtual interventions. *Cardiovascular Engineering and Technology* 6, 281–293. doi:10.1007/s13239-015-0218-x

Davidson, L. (2009). Large Eddy Simulations: How to evaluate resolution. *International Journal of Heat and Fluid Flow* 30, 1016–1025. doi:10.1016/j.ijheatfluidflow.2009.06.006
